# SHP2 inhibition enhances the anticancer effect of Osimertinib in EGFR T790M mutant lung adenocarcinoma by blocking CXCL8 loop mediated stemness

**DOI:** 10.1186/s12935-021-02056-x

**Published:** 2021-07-03

**Authors:** Leiming Xia, Fan Yang, Xiao Wu, Suzhi Li, Chen Kan, Hong Zheng, Siying Wang

**Affiliations:** 1 Basic College of Medicine, Anhui Medical University, 81 Meishan road, Hefei, Anhui China; 2 Department of Hematology, The Third affiliated hospital of Anhui Medical University, Hefei, China; 3 Department of Hematology, The fourth affiliated hospital of Anhui Medical University, Hefei, China; 4 Laboratory Center for Medical Science Education, Anhui Medical University, Hefei, China

**Keywords:** SHP2, T790M mutation, Lung adenocarcinoma, CXCL8 feedback loop, Stemness, Osimertinib

## Abstract

**Background:**

Additional epidermal growth factor receptor (EGFR) mutations confer the drug resistance to generations of EGFR targeted tyrosine kinase inhibitor (EGFR-TKI), posing a major challenge to developing effective treatment of lung adenocarcinoma (LUAD). The strategy of combining EGFR-TKI with other synergistic or sensitizing therapeutic agents are considered a promising approach in the era of precision medicine. Moreover, the role and mechanism of SHP2, which is involved in cell proliferation, cytokine production, stemness maintenance and drug resistance, has not been carefully explored in lung adenocarcinoma (LUAD).

**Methods:**

To evaluate the impact of SHP2 on the efficacy of EGFR T790M mutant LUAD cells to Osimertinib, SHP2 inhibition was tested in Osimertinib treated LUAD cells. Cell proliferation and stemness were tested in SHP2 modified LUAD cells. RNA sequencing was performed to explore the mechanism of SHP2 promoted stemness.

**Results:**

This study demonstrated that high SHP2 expression level correlates with poor outcome of LUAD patients, and SHP2 expression is enriched in Osimertinib resistant LUAD cells. SHP2 inhibition suppressed the cell proliferation and damaged the stemness of EGFR T790M mutant LUAD. SHP2 facilitates the secretion of CXCL8 cytokine from the EGFR T790M mutant LUAD cells, through a CXCL8-CXCR1/2 positive feedback loop that promotes stemness and tumorigenesis. Our results further show that SHP2 mediates CXCL8-CXCR1/2 feedback loop through ERK-AKT-NFκB and GSK3β-β-Catenin signaling in EGFR T790M mutant LUAD cells.

**Conclusions:**

Our data revealed that SHP2 inhibition enhances the anti-cancer effect of Osimertinib in EGFR T790M mutant LUAD by blocking CXCL8-CXCR1/2 loop mediated stemness, which may help provide an alternative therapeutic option to enhance the clinical efficacy of osimertinib in EGFR T790M mutant LUAD patients.

**Supplementary Information:**

The online version contains supplementary material available at 10.1186/s12935-021-02056-x.

## Introduction

Lung cancer is one of the primary causes of cancer related incidence and mortality worldwide [1], and lung adenocarcinoma (LUAD) is the most prevalent histological subtype [2–4]. The characterization of LUAD is more precise nowadays pointing to gain-of-function mutations on epidermal growth factor receptor (EGFR) [5]. EGFR mutation plays a critical role in regulating the malignancy of lung cancer cells, and East Asian LUAD patients are harboring the highest mutation rate of EGFR [6–9].

Generations of mutant EGFR targeted tyrosine kinase inhibitors (EGFR-TKIs) have impressively improved the clinical outcome of LUAD patients [10, 11]. However, additional T790M mutation confers half of the acquired resistant cases treated under the first-generation EGFR-TKIs that occur in 8–14 months [12–15]. Furthermore, updated data reported that around 43.16 % T790M mutant patients are resistant to third-generation TKI, Osimertinib, which is designed to specifically target the ATP site of EGFR with T790M mutation [16–18]. Thus, exploring effective alternative approaches is therapeutically needed for the treatment of T790M mutant LUAD patients. Therapies combined with Osimertinib were currently preferred which have been shown to significantly inhibit tumor progression, and possibly reverse Osimertinib resistance [19, 20].

Cancer stem cells (CSCs) are characterized as a cancer cell sub-population endowed with the capacity of tumor initiation [21], disease recurrence [22], cancer dissemination [23–25], as well as aggressive drug resistance [26, 27]. CD133 was identified as an important stemness marker for LUAD [28], and CSCs targeting therapies have been reported to potentially reverse the drug resistance [29]. SHP2 (Src homology 2-containing phosphotyrosine phosphatase 2) plays a critical role in regulating CSCs [30, 31] and cytokine release [32–34]. The feedback loop of secreted cytokines plays an important role in maintaining the stemness of progenitor cells [35–37]. CXCL8 promotes the formation of tumor micro-environment (TME) [38] and the enrichment of CSCs [39–41]. More importantly, the feedback loop of secreted CXCL8 facilitates the re-population of drug resistant cancer cells that exacerbates the clinical outcome in multiple cancer types [42, 43], and such resistance could be reversed with CXCL8 inhibition [43].

It is currently not clear whether SHP2 regulated cancer stemness contributes to the drug resistance to EGFR-TKIs. In this study we show that SHP2 inhibition sensitizes the T790M mutant LUAD to respond to Osimertinib treatment *via* stemness suppression by blocking the autocrine CXCL8 feedback loop, and thus improve the prognosis of EGFR T790M mutant LUAD patients.

## Materials and methods

### Cell lines and reagents

The PC9 (Del19) and PC9GR (harboring T790M&Del19 mutation) LUAD cell lines were generously gifted by Dr. Changzhi Xu (Anhui University, Hefei, China). Both PC9 and PC9GR cells were cultured in Dulbecco’s Modified Eagle Medium (DMEM) supplemented with 10 % Fetal Bovine Serum (FBS), 100 U/mL penicillin and 100 mg/mL streptomycin, and incubated in 5 % CO_2_ at 37 ℃.

Lv-SHP2, Lv-SHP2-RNAi (RNA interference) and scrambled controls were purchased from Shanghai Genechem Co.,Ltd. Recombinant human Epidermal Growth Factor (hEGF) (SRP3027) and Fibroblast Growth Factor (hFGF) (GF003AF) were purchased from Sigma-Aldrich. SHP2 inhibitor (SHP099, Cat#: HY-100,388), recombinant human CXCL8 (Cat#: HY-P7224), CXCR2 inhibitor (Danirixin, Cat#:HY-19,768), CXCR1/2 inhibitor (Reparixin, CAT#: HY-15,251) and Osimertinib (AZD-9291, Cat#: HY-15,772) were bought from MCE LLC. Human CXCL8 Immunoassay Quantikine ELISA Kit (Cat#:D8000C) was purchased from R&D Systems.

Antibodies against SHP2(Clone:D50F2, Cat#:3397), CD133(Clone:D2V8Q, Cat#:64,326), GSK3β(Clone:D5C5Z, Cat#:12,456), p-GSK3β(ser9) (Clone:5B3, Cat#:9323), β-Catenin(Clone:D10A8, Cat#:8480), ERK1/2(Clone:137F5, Cat#:4695), p-ERK1/2(Thr202/Tyr204) (Clone:20G11, Cat#:4376), AKT(Clone:11E7, Cat#:4685), p-AKT(Ser473) (Clone:D9E, Cat#:4060), p-RelA/p65(Ser536) (Clone:93H1, Cat#:3033), p-IkBβ(Thr19/Ser23) (Cat#:4921), Ki67(Clone:D2H10, Cat#:9027) and β-actin (Clone:8H10D10, Cat#:3700) were purchased from Cell Signaling Technology. For Flow Cytometry, anti-CD133 monoclonal antibody conjugated with Super Bright 436 (Clone:TMP4, Cat#:62-1338-42), and mouse IgG1 kappa Isotype Control (Cat#:62-4714-42) were obtained from eBioscience.

### Lentiviral transduction

Lentiviral transduction was performed following user’s protocol using GV298 plasmids encoding over-expression (Ubi-MCS-3FLAG-CBh-gcGFP-IRES-puromycin) and RNAi (U6-MCS-Ubiquitin-Cherry-IRES-puromycin) against SHP2 and homologous control. Briefly, 1 × 10^5^ cells were seeded in 6 well plate and incubated overnight. 20µL of 1 × 10^8^ TU/mL lentivirus diluted in 1 mL complete DMEM medium was added into each well, and medium changed after incubation for another 12 h. Fluorescence expression was used for monitoring the efficacy of transfection. Infected PC9 and PC9GR cells were selected by puromycin (0.5 µg/ml) for 7 days to generate stable clones and maintained in 0.3 µg/mL of puromycin condition for over 1 month. The transfected cells were defined as LV-SHP2 for SHP2 overexpressing cells and LV-SHP2-RNAi for SHP2 knock-down cells, respectively.

### Transcriptome analyses based on next-generation sequencing (NGS)

In order to analyze the relevant gene transcription of SHP2 modification in LUAD, the NGS of lentivirus modified and parental PC9GR cells were performed by the Beijing Genome Institute (BGI, Shenzhen, China). Total RNA was extracted, mRNA was enriched and then the cDNA libraries were prepared. Transcriptome expression were generated by equal quantities of RNA from lentivirus modified and parental PC9GR cells, 3 biological duplications were repeated in each group. Bowtie2 (v2.2.5) was applied to align the clean reads to the gene set, and based on a database for this organism built by BGI, in which all known and novel coding transcripts were included, expression level of genes ware calculated by RSEM (v1.2.12). The heatmap was then generated by pheatmap (v1.0.8) according to gene expression profiles in different samples. To gain further insight into phenotype changes, KEGG (https://www.kegg.jp/) enrichment analysis was performed by Phyper based on Hypergeometric test. The significant levels of terms and pathways were corrected by Q value with a rigorous threshold (Q value ≤ 0.05) by Bonferroni.

### Bioinformatics analysis

The Kaplan–Meier analysis of the overall survival (OS) and progression free survival (PFS) of all 719 LUAD patients with different SHP2 and other EGFR downstream genes expression levels were performed online using the Kaplan–Meier Plotter (http://kmplot.com/analysis/) with auto-selected best cutoff of patients grouping for statistical significance set to *p* < 0.001 [44].

### Cell Viability testing by MTT assay

Cell Viability was tested by MTT assay. Briefly, 2 × 10^3^ cells were seeded in each well of 96-well plates with 200µL medium and incubated for time indicated in the text. 50 µl of MTT solution was added to the bottom of wells and supernatant discarded after 4 h of incubation, MTT formazan crystal was then dissolved in dimethylsulfoxide (DMSO), and absorbance was measured by a Multi-Mode Microplate Reader (Varioskan Flash, Thermo Scientific) at a wavelength of 490 nm.

### Colony formation

For soft agar colony formation testing, 1.5 ml of 0.6 % agarose was filled into the wells of 6-well plate and solidified in fume hood for 30 min. lentivirus transduced and parental PC9, PC9GR cells were washed with phosphate buffered saline (PBS) and 2000 cells for each sample were suspended in 0.35 % agarose in DMEM supplemented with 10 % FBS and plated on top of the bottom agarose. Clones were scored after 2 weeks of incubation.

For colony formation assays, lentivirus transduced PC9, PC9GR and parental cells were washed with PBS and plated at a cell density of 200 cells per well in DMEM supplemented with or without treatment, and clones were fixed with crystal violet and scored after 2 weeks of incubation.

### Tumorsphere formation

For tumorsphere formation assays, lentivirus transduced and parental PC9, PC9GR cells were seeded in 1.2 % agarose pre-coated 6-well plates at a density of 2000 cells per ml in serum free DMEM/F12 medium (Gibco) with 10 ng/mL bFGF, 20 ng/mL EGF. The culture medium was changed every 2 days until the sphere generated. After 7 to 10 days of culturing, the spheres were collected for further experiments.

### Flow cytometry testing

1 × 10^6^ cells/100µL was aliquoted into FACS tubes. 5µL anti-CD133 antibody was added into each tube and vortexed. And then the mix was incubated for 30 min on ice in the dark. After centrifuging the suspended cells at 300×*g* for 5 min, the supernatant was drawn out and cells resuspended with 2 mL of washing buffer. The washing was repeated twice, followed by resuspending the cells in 400µL of buffer for analysis (BD Bioscience, San Jose, CA, USA). Data was analyzed in FlowJo Software version 7.6 (Tree Star, Inc., Ashland, OR).

### Elisa assay

For ELISA analysis, 5 × 10^4^ lentivirus transduced PC9, PC9GR and parental cells were seeded in 6-well plates for 48 h. Culture supernatant was collected to quantify the secreted CXCL8 using pre-coated ELISA kit according to the manufacturer’s protocol. Briefly, prepared CXCL8 diluted standard solution, sample or control (100 µL) was added to each well, and incubated at room temperature for 2 h. After washing each well for four times with washing buffer, 100 µL of CXCL8 conjugate was added into each well and incubated for 1 h at room temperature. After four washes, 200 µL of substrate solution was added to each well and the plate was incubated for 30 min at room temperature in the dark. 50µL of stop solution was added to each well, and the optical density of each well was determined using a microplate reader set to 450 nm. A standard curve was created for calculating the concentration of secreted CXCL8.

### Western blotting

Cell were lysed in protein lysis buffer containing protease and phosphatase inhibitors. Protein concentration was determined by the BCA Protein Assay Kit. Equal amount of proteins was separated using electrophoresis by SDS-PAGE gels, and transferred to PVDF membrane. After blocking in 5 % milk, PVDF membranes were incubated with specific antibodies against targeted molecules, respectively. The bands were detected with enhanced chemiluminescence and quantified by Image J software.

### Immunohistochemistry (IHC)

For IHC examination, the tumors from experimental groups were harvested, fixed and embedded in paraffin and further examined for the expression of indicated proteins. Briefly, paraffin-embedded slides were rehydrated and antigen retrieved. Endogenous peroxidase was quenched by treatment with 3 % hydrogen peroxide for 5 min. CD133, Ki67 were stained following the manufacturer`s protocol.

### Animal ethics

Six–8 weeks old balb/c nude mice were obtained from GemPharmtech Co., Ltd. (Nanjing, Jiangsu) and maintained in SPF animal room with a positive pressure containment rack. For tumor models, mice were euthanized when the tumor reached the humane endpoints, and 2.5 L/min CO2 was used to euthanize the animals for 5-7 min. The animal experimental protocols were approved by the Anhui Medical University Animal Care & Use Committee and conducted in accordance with the guideline for laboratory animal usage of Anhui Medical University.

### Tumorigenicity of SHP2 modified LUAD tumor cells in gradient concentration

Tumor cells were suspended at indicated concentration (5 × 10^4^, 5 × 10^3^ and 5 × 10^2^ cells), and mixed with Matrigel (BD Biosciences) at the ratio of 1:1, and injected into the lower flanks of 5 weeks Balb/c-Nude mice subcutaneously. The mice were monitored 2–3 times per week until tumor formation.

### Xenograft model

For testing the sensitivity of PC9 and PC9GR cells to Osimertinib, 1 × 10^7^ cells were injected subcutaneously into the lower flanks of healthy 5 weeks old nude mice. Mice bearing the same size of tumors were randomly grouped and treated with DMSO or Osimertinib (5.0 mg/kg daily by oral gavage, subcutaneous (s.c.) injection 2 times per week for 3 weeks), treatment began in the second week post tumor injection. Mice were monitored 2–3 times per week, and tumor size was evaluated by formula: [length × (width)^2^]/2. Mice were sacrificed at the end of 49 days post injection, and tumors harvested afterward.

For testing the sensitivity of SHP2 high and low expressing PC9GR cells to Osimertinib. 1 × 10^7^ cells were injected subcutaneously into the lower flanks of healthy 5 weeks old nude mice. Mice bearing the same size of tumors were randomly grouped and treated with DMSO or Osimertinib (5.0 mg/kg daily by oral gavage, s.c., 2 times per week for 3 weeks), treatment began in the second week post tumor injection. The mice were monitored 2–3 times per week, and tumor size was evaluated by formula: [length × (width)^2^]/2. Mice were sacrificed at the end of 41 days post injection, and tumors harvested afterward.

To identify the tumor formation ability of PC9GR Lv-SHP2 and Lv-SHP2RNAI cells with blockade of CXCL8-CXCR1/2, 1 × 10^7^ cells were collected in PBS and injected subcutaneously into the lower flanks of healthy 5 weeks old nude mice. Mice bearing the same size of tumors were randomly separated into three groups, DMSO, Danirixin (25 mg/Kg, s.c., 3 times per week for 3 weeks) and Reparixin group (30 mg/kg, s.c., 3 times per week for 3 weeks), the treatment began in the second week post injection. Mice were sacrificed at the end of 41 days post injection, and tumors harvested afterward.

### Statistical analysis

Data in the current experiment were represented by means ± SEM. For comparisons between two groups, statistical significance was performed by Student’s t-test, For comparisons of more than two groups, statistical significance was performed by ANOVA. Statistical analysis was performed with GraphPad Prism 7. *p* values of < 0.05 are considered statistically significant.

## Results

### Higher expression of SHP2 indicates poor survival and Osimertinib resistance in LUAD patients

**Fig. 1:**
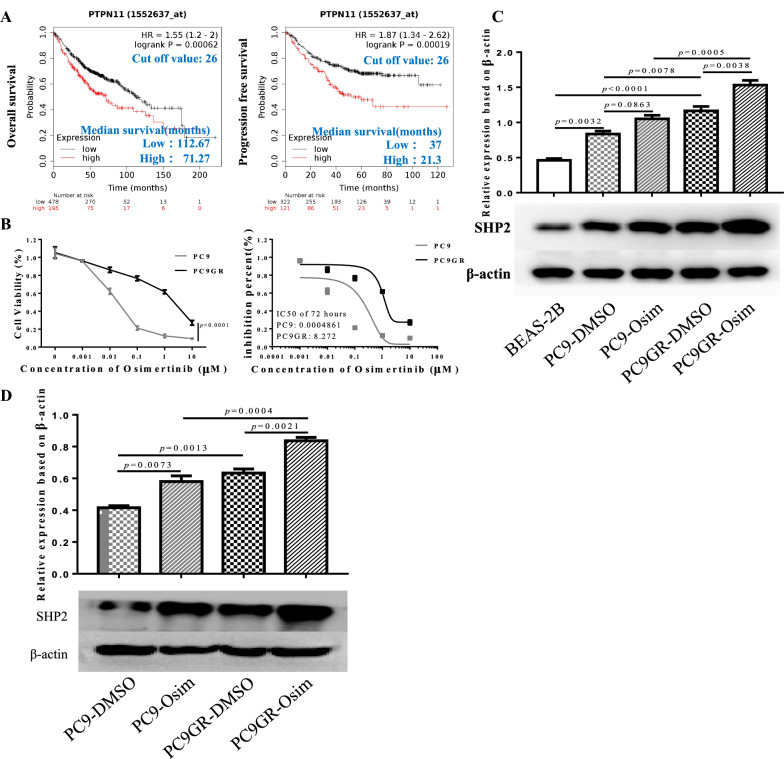
Higher expression of SHP2 indicates poor survival and Osimertinib resistance in LUAD patients. **A** High SHP2-expressing LAUD patients suffered poor OS (71.27 months vs. 112.67 months, p = 0.00062) and PFS (21.3 months vs. 37 months, p = 0.00019) than low SHP2-expressing patients. **B** The IC50 of PC9 and PC9GR cells to Osimertinib treated for 72 h were 0.0004861 and 8.272, respectively. **C** LUAD cancer cells express high level SHP2 protein than BEAS-2B pulmonary epithelial cells, and PC9GR cells harboring higher SHP2 than PC9 cells. More interestingly, SHP2 was enriched in Osimertinib treated PC9GR and PC9 cells compared with corresponding control cells. **D** Higher SHP2 protein expressed in the tumor of Osimertinib treated tumor than that of DMSO group. Each experiment was repeated 3 times

To determine whether SHP2 participates in regulating disease progression and Osimertinib resistance in LUAD patients, we first assessed the prognostic differences of LUAD patients harboring distinct levels of SHP2 mRNA. Combined dataset (data from TCGA, GSE and CAARRAY) including 2437 LUAD patients were analyzed using the online tool (http://kmplot.com/analysis/) [44]. Analysis showed that high SHP2 mRNA expression of LAUD patients suffered poor overall survival (OS) (71.27 months vs. 112.67 months, *p* = 0.00062) and progression free survival (PFS) (21.3 months vs. 37 months, *p* = 0.00019) compared to patients expressing low levels of SHP2 mRNA, suggesting that SHP2 might be involved in the tumor progression of LUAD (Fig. 1A). Meanwhile, the activity of downstream key molecules of EGFR signaling were altered suggesting SHP2 involves in the regulation. Generally, we found high mRNA expression levels of genes in MEK-ERK and PI3K-AKT pathways were also associated with poor outcome of LUAD (Additional file 1: Fig. S1). Next, we tested whether SHP2 expression is associated with Osimertinib sensitivity in LUAD cells. We investigated the half maximal inhibitory concentration (IC50) of PC9 and PC9GR cells respectively, and tested the SHP2 protein in the remaining live cells treated with Osimertinib at IC50 (Fig. 1B). Here, we report that SHP2 expression was much higher in PC9GR cells than PC9 cells, and malignant LUAD cells contained higher SHP2 than BEAS-2B pulmonary epithelial cells. Also, SHP2 was enriched in Osimertinib treated PC9GR and PC9 cells at IC50, meaning that SHP2 might mediate Osimertinib resistance in T790M mutant LUAD cells (Fig. 1 C). Further, we generated PC9 and PC9GR tumor bearing mice and found that SHP2 protein level in Osimertinib treated tumor was higher than that in the DMSO treated group (Fig. 1D). Hence, we report that high SHP2 expression levels are related to poor outcome of LUAD patients, and SHP2 expression was enriched in Osimertinib resistant T790M mutant LUAD cells.

### SHP2 reduce the sensitivity of T790M mutant LUAD cells to Osimertinib

**Fig. 2:**
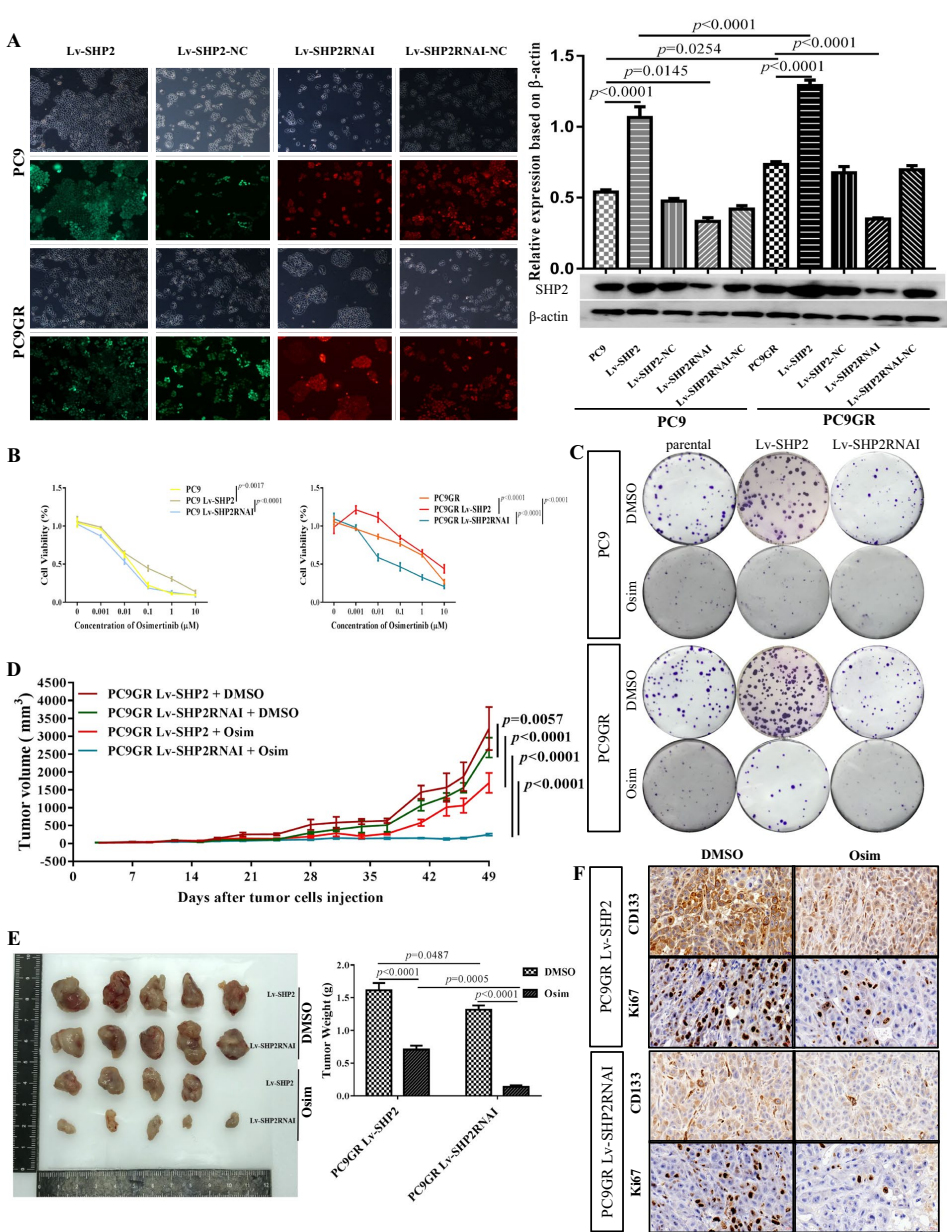
SHP2 reduce the sensitivity of T790M mutant LUAD cells to Osimertinib. **A** Lentiviral transfected LUAD cells were generated to modify SHP2 expression, Lv-SHP2 plasmids were labeled in green with GFP reporter expression, Lv-SHP2RNAI plasmids in red with mCherry reporter expression, and SHP2 expression was confirmed by western blot (right panel). **B** The viability of PC9 and PC9GR cells with over-expressed SHP2 were significantly higher than that of their parental cells in a dose dependent manner, while the viability of SHP2 knock-down cells were significantly lower than that of their parental cells. **C** High SHP2-expressing PC9 and PC9GR cells formed substantially larger residual crystal violet than that of their parental cells when co-cultured with Osimertinib, while the residual crystal violet generated by SHP2 knock-down cells was obviously decreased. **D** Osimertinib dramatically shrunk the tumor size of PC9GR tumor *in vivo*. **E**, Osimertinib significantly reduced the tumor size of SHP2 inhibited PC9GR cells than SHP2 over-expressing PC9GR cells. F, the proportion of Ki67 and CD133 positive CSCs was much higher in LV-SHP2 PC9GR tumor than in LV-SHP2RNAI tumor of Osimertinib administration. Each experiment was repeated 3 times

To examine SHP2’s potential function in mediating Osimertinib resistance of T790M mutant LUAD, we tested the response of SHP2 modified LUAD cells to Osimertinib treatment. First, stable SHP2 overexpressing LUAD cells were generated using lentiviral plasmids harboring SHP2 (Lv-SHP2), and SHP2 knockdown was achieved using RNA interference (Lv-SHP2RNAi) in both PC9 and PC9GR cells. Lv-SHP2 plasmids were labeled in green, and Lv-SHP2RNAi plasmids in red, and immunofluorescence results confirmed that SHP2 modified cells were established successfully (Fig. 2A). These stably infected cells with modified SHP2 expression were further used for both in vitro and in vivo studies to investigate the role of SHP2 in mediating Osimertinib sensitivity. First, we investigated the relationship between SHP2 and Osimertinib sensitivity in T790M mutant LUAD cells. After co-culturing with Osimertinib for 72 h, the viability of PC9 and PC9GR cells with high SHP2 were significantly higher than that of their parental cells in a dose dependent manner, while the SHP2 knock-down cells significantly lower than their parental cells (Fig. 2B). High SHP2 expressing PC9 and PC9GR cells formed substantially larger residual crystal violet than that of their parental cells when co-cultured with Osimertinib, while the size of residual crystal violet generated by SHP2 knock-down cells was clearly decreased (Fig. 2C, Additional file 2: Fig. S2A). Therefore, in vitro data showed that PC9GR cells with higher SHP2 expression were more resistant and showed higher proliferation to in response to Osimertinib treatment than their parental cells, while SHP2 inhibition makes PCRGR cells less resistant. Furthermore, in vivo data showed that Osimertinib dramatically shrunk the PC9GR tumor size (Fig. 2D). On average, Osimertinib reduced 55.96 % of the tumor weight of SHP2 over-expressing tumors from 1.612 to 0.710 g in control, and 89.51 % of the tumor weight from 1.316 to 0.138 g in SHP2 knock-down tumors (Fig. 2E). In IHC assay, the percentage of Ki67 positive cells in LV-SHP2 RNAi PC9GR tumor was smaller than LV-SHP2 PC9GR tumor when treated with Osimertinib. More importantly, the proportion of CD133 positive CSCs was much higher in LV-SHP2 PC9GR tumors than that in LV-SHP2RNAI tumors in response to Osimertinib treatment (Fig. 2F, Additional file 2: Fig. S2B). Therefore, SHP2 overexpressing PC9GR cells showed significantly higher resistance to Osimertinib than SHP2 knock-down cells through enhancement of cell proliferation and CSCs enrichment.

### SHP2 mediates the malignant proliferation of LUAD cells

**Fig. 3:**
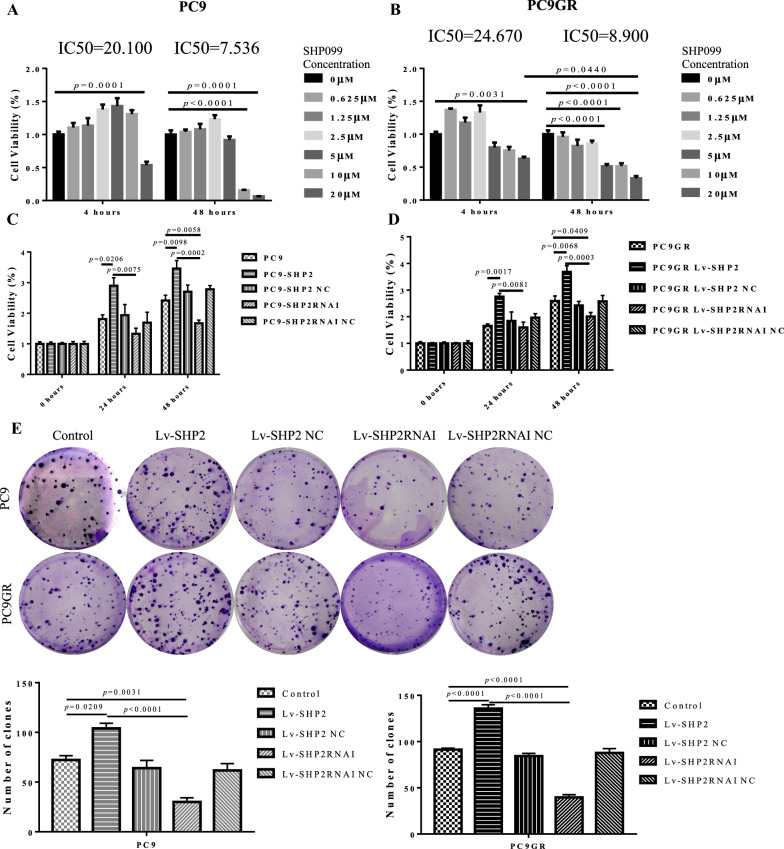
SHP2 inhibition suppressed the proliferation of LUAD cells. **A** The IC50 of SHP099 in PC9 cells was 20.100 and 7.536 at 4 and 24 h, respectively. **B** The IC50 of SHP099 in PC9GR cells was 24.670 and 8.900 at 4 and 24 h, respectively. **C**, **D** SHP2 over-expressing PC9GR and PC9 cells showed aggressive proliferation than SHP2 knock-down cells after 24 and 48 h. E, colony formation assay showed that larger residual clones were established by PC9 and PC9GR cells with high expression of SHP2 compared with parental cell lines, while smaller residual clones were established in PC9 and PC9GR cells with low expression of SHP2. Each experiments was repeated 3 times

To test whether SHP2 mediates the malignant proliferation of LUAD cells, we first analyzed the cell viability of parental and SHP2 lentivirus transfected PC9 and PC9GR cells treated with SHP2 inhibitor-SHP099, the data showed that SHP099 significantly inhibited the proliferation of PC9 and PC9GR cells in a dose dependent manner. We found that the IC50 of SHP099 in PC9 cells was 20.100 and 7.536 at 4 and 24 h (Fig. 3 A), and in PC9GR cells was 24.670 and 8.900 at 4 and 24 h respectively (Fig. 3B). Further, we evaluated SHP2 derived proliferation in PC9 and PC9GR cells. SHP2 overexpressing PC9GR and PC9 cells showed aggressive proliferation than SHP2 knock-down cells after 24 or 48 h of culture (Fig. 3C, D). Similarly, colony formation assay showed that larger residual clones were established by PC9 and PC9GR cells overexpressing SHP2 compared with their parental cells, while even smaller residual clones were derived in PC9 and PC9GR cells with SHP2 knock-down (Fig. 3E). These data upheld the role of SHP2 in regulating the proliferation of LUAD with EGFR T790M mutation (Fig. 2), with potentially a parallel change in the stemness of CD133^+^ CSCs which needs further validation.

### SHP2 mediates the stemness and function of LUAD cells

**Fig. 4:**
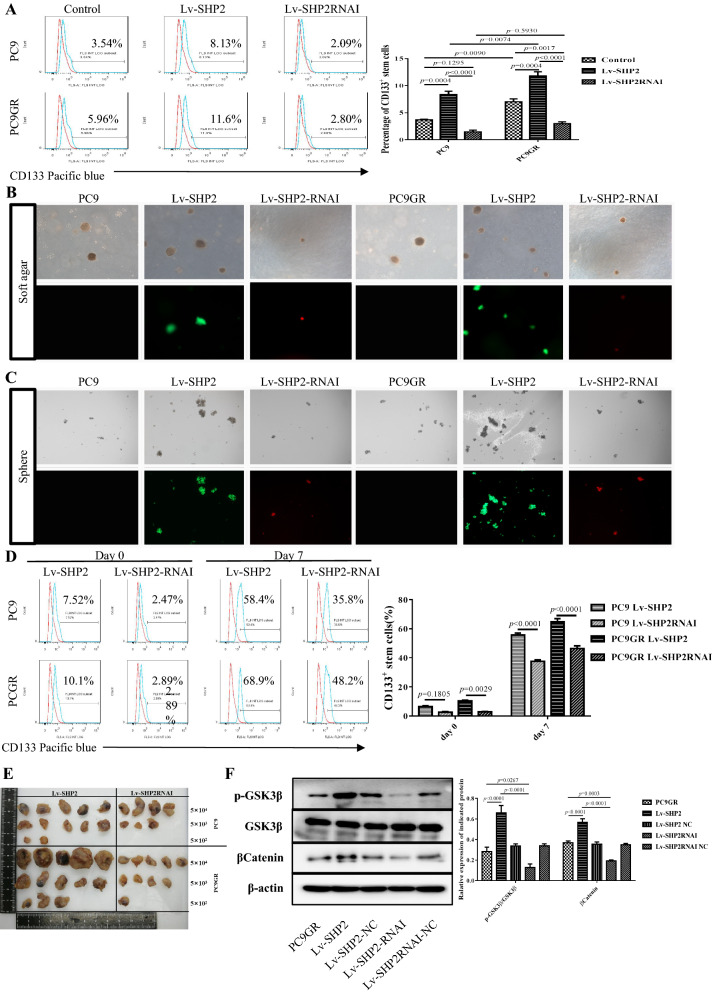
The stemness of LUAD cells were damaged by SHP2 inhibition.** A** The percentage of CD133^+^ CSCs increased from 7.013 ± 0.560 % of parental PC9GR cells to 11.767 ± 0.801 % of SHP2 over-expressed cells, while, the proportion of CD133^+^ CSCs decreased to 2.973 ± 0.346 % in SHP2 knock down cells. Meanwhile, CD133^+^ LUAD CSCs in SHP2 over-expressed PC9 cells increased from 3.697 ± 0.092 % of parental cells to 8.370 ± 0.607 %, while the proportion of CD133^+^ CSCs decreased to 1.467 ± 0.319 % when knocking down SHP2. **B** PC9 and PC9GR cells with high SHP2 formed bigger and more spheres than homologous parental cells in soft Agar culturing, while low SHP2 expressed cells showed impaired ability to form spheres. **C** PC9 and PC9GR cells with high SHP2 formed bigger and more spheres than their parental cells, while low SHP2 expressed PC9 and PC9GR cells had a significantly attenuated ability to form spheres. **D** After 7 days of culture, the proportion of CSCs in SHP2 high-expressing PC9GR cells was significantly enriched from 10.410 ± 0.506 % of the parental cells to 64.833 ± 2.188 %, and the CSCs of low SHP2 expressed PC9GR cells was also significantly enriched after 7 days which increased from 2.910 ± 0.260 % of the parental cells to 46.267 ± 1.933 %. **E** 50,000, 5000 and 500 high and low SHP2 modified PC9 and PC9GR cells were inoculated into Balb/c-Nu nude mice, respectively, and the SHP2 cells showed aggressive capability of generating tumors. **F** Phosphorylated protein of GSK3β and βCatenin were reduced in low SHP2 PC9GR cells. Each experiment was repeated 3 times

To further test whether SHP2 is involved in regulating CSCs of LUAD that promotes malignancy, we evaluated the stemness of SHP2 modified cells. We found that the percentage of CD133^+^ CSCs increased from 7.013 ± 0.560 % in parental PC9GR cells to 11.767 ± 0.801 % in SHP2 over-expressing cells, while decreased to 2.973 ± 0.346 % in SHP2 knock-down cells. Meanwhile, the percentage of CD133^+^ LUAD CSCs in SHP2 over-expressing PC9 cells increased to 8.370 ± 0.607 % from 3.697 ± 0.092 % in parental cells, while decreased to 1.467 ± 0.319 % when knock down SHP2 (Fig. 4A). We further tested the role of SHP2 in mediating the formation of tumor spheres from CSCs in LUAD. In soft agar culturing, PC9 and PC9GR cells with high SHP2 formed bigger and more spheres than homologous parental cells, while low SHP2 expressing cells formed smaller and fewer spheres showing an impaired ability of sphere formation (Fig. 4B). Similarly, in sphere formation, PC9 and PC9GR cells with high SHP2 expression forming bigger and more spheres than their parental cells, while low SHP2 expressing PC9 and PC9GR cells showed a significantly attenuated ability to form spheres (Fig. 4C). Further, we examined the proportion of CD133^+^ cells after culturing in order to confirm bigger spheres generated by SHP2 overexpressing cells was indeed derived by CSCs accumulation. After 7 days of in vitro sphere culturing, the proportion of CSCs in SHP2 overexpressing PC9GR cells significantly enriched from 10.410 ± 0.506 % to 64.833 ± 2.188 %, and the CSCs of SHP2 knock-down PC9GR cells also significantly enriched from 2.910 ± 0.260 % of to 46.267 ± 1.933 %. Similar results were also obtained in PC9 cells (Fig. 4D and Additional file 3: Fig. S3A). In addition, we tested the ability of tumorigenesis for SHP2 modified LUAD cells. A gradient of 50,000, 5000 and 500 cells were inoculated into Balb/c-Nu nude mice, and the SHP2 overexpressing cells showed more aggressive tumor generation compared with SHP2 knock-down cells in both PC9 and PC9GR background (Fig. 4E), with an accelerated rate of tumorigenesis (Additional file 4: Table S1). For elucidating the molecular mechanism, we performed transcriptome sequencing in SHP2 modified PC9GR cells and 1,203 differentially expressed genes were identified for clustering which further highlighted the stem cell pathway (Additional file 3: Fig. S3B–D). Immunoblotting analysis of the GSK3β-β-Catenin pathway revealed that phosphorylated GSK3β and β-Catenin were readily increased in SHP2 overexpressing PC9GR cells and reduced in SHP2 knock-down cells (Fig. 4F). Therefore, these results conclude that SHP2 mediates Osimertinib resistance through enhancing the proportion and function of CSCs in LUAD.

### SHP2 facilitates CXCL8 secretion of EGFR T790M mutant LUAD

**Fig. 5:**
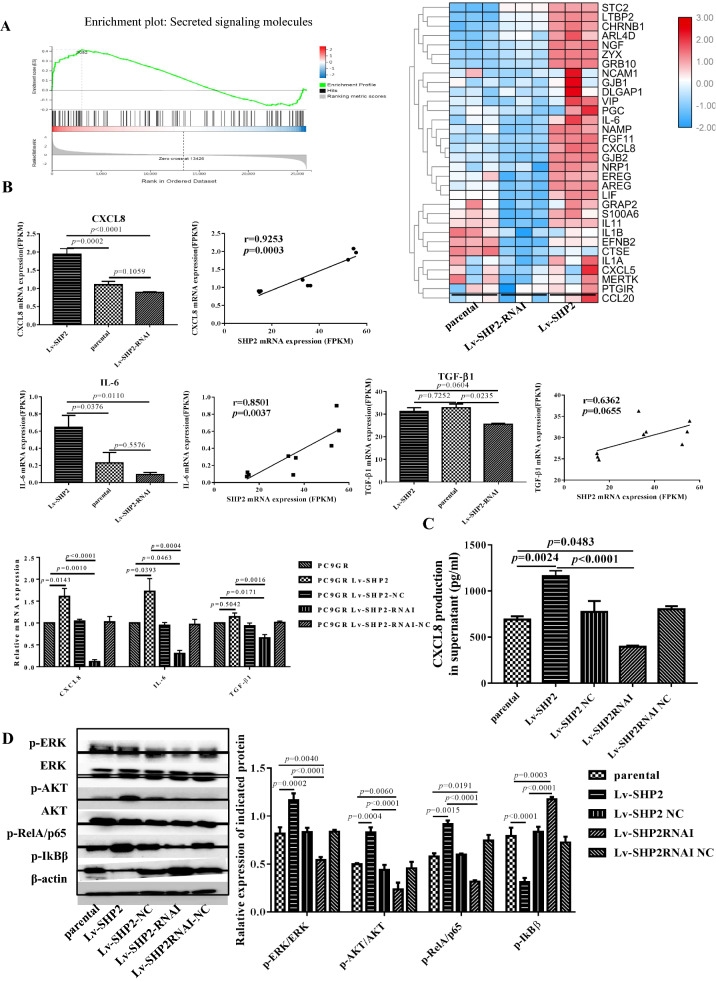
SHP2 facilitates the CXCL8 secretion of EGFR T790M mutant LUAD. **A** Differentially expressed mRNA in inhibited, over-expressed and parental PC9GR cells in plot of secreted signaling molecule, a subset of 32 significant genes were identified with an FDR of < 0.05. **B** mRNA expression of CXCL8 and IL-6 genes showed significant diversity among the three groups, while TGF-β1 mRNA was not significantly altered. Meanwhile the correlation analysis implied a significant correlation coefficient of r = 0.9253 between CXCL8 and SHP2, the relationship of IL-6 and SHP2 was conferred a r = 0.8501. For NGS data analysis, three replicates for each biological group were included in the analysis. **C** The concentration of CXCL8 in the culture medium of SHP2 over-expressing PC9GRcells was significantly higher than that of parental cells, while the CXCL8 in the supernatant of SHP2 inhibited PC9GR cells was significantly reduced. **D** Activated ERK, AKT and RelA/p65 were significantly up-regulated in SHP2 over-expressing cells than parental cells, and down-regulated in SHP2 knock-down cells. Each experiment was repeated 3 times

SHP2 promoted the tumor cell stemness in EGFR T790M mutant LUAD, and secreted cytokines in the tumor microenvironment (TME) were essential for the stemness regulation and maintenance [45, 46]. To further identify key cytokines responsible for SHP2 mediated maintenance of CSCs, we performed enrichment analysis for secreted signaling molecule based on transcriptome data, a set of 32 differentially expressed genes were identified including CXCL8 (Fig. 5A and Additional file 5: Table S2). We further examined CXCL8, IL-6 and TGF-β1, which are considered to be related to the regulation and maintenance of CSCs. CXCL8 and IL-6 mRNA expression showed significant difference among the three groups, while TGF-β1 mRNA was not significantly altered. Meanwhile, correlation analysis implied a significant correlation coefficient of r = 0.9253 between CXCL8 and SHP2, the relationship of IL-6 and SHP2 was conferred a r = 0.8501 (Fig. 5B). We then measured the concentration of CXCL8 in the supernatant of parental and SHP2 modified PC9GR cells. SHP2 overexpressing PC9GRcells contains a significantly higher concentration of secreted CXCL8 in the medium than their parental cells, while SHP2 knock-down PC9GR cells contains a significantly reduced level of secreted CXCL8 (Fig. 5C). To illustrate the molecular mechanism of SHP2 mediated cytokine secretion, we classified the transcriptome expression and found that differentially expressed genes were mostly enriched in MAPK and stem cell pathways (Additional file 3: Fig. S3). We further monitored EGFR downstream NF-κB signaling. The activated/phosphorylated ERK, AKT and RelA/p65 were significantly upregulated in SHP2 overexpressing cells than their parental cells, and downregulated in SHP2 knock-down cells (Fig. 5D). These data suggest that SHP2 plays a critical role in mediating CXCL8 secretion of LUAD via ERK-AKT-NFκB pathway.

### SHP2 mediates the stemness and tumorigenesis of EGFR T790M mutant LUAD through a CXCL8/CXCR1 positive feedback loop

**Fig. 6:**
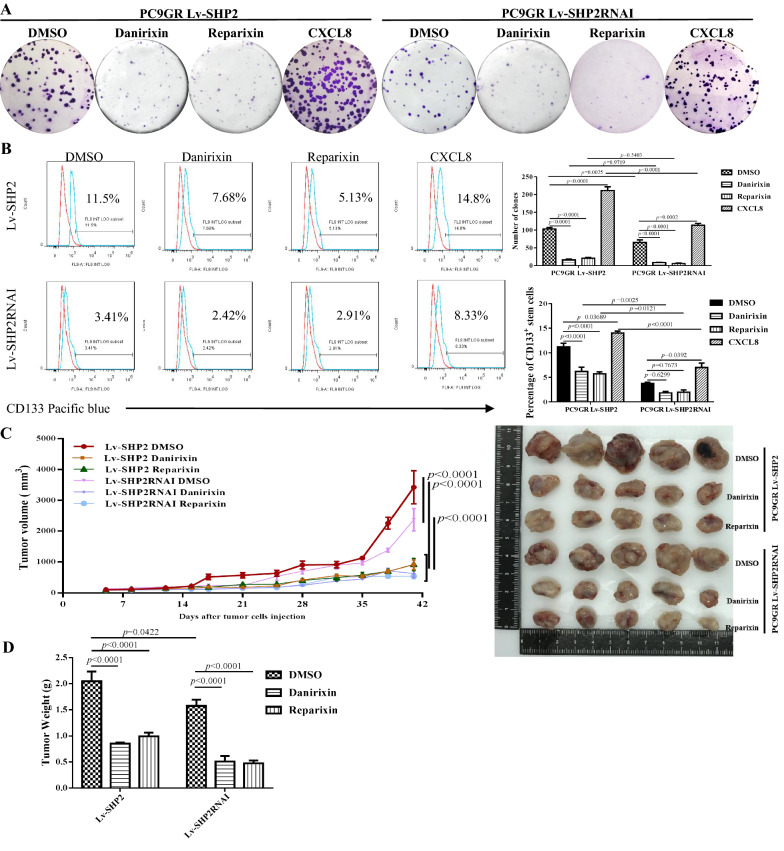
SHP2 inhibition suppresses the stemness and tumorigenesis of EGFR T790M mutant LUAD by CXCL8/CXCR1 positive feedback loop. **A** The clones were dramatically inhibited by Danirixin or Reparixin, while exogenous CXCL8 significantly enhanced the colony formation of PC9GR. **B** The percentage of CSCs was reduced in the setting of CXCL8-CXCR1/2 loop blockage, and recombinant CXCL8 could reverse the CSCs proportion. **C** In SHP2 over-expressed panel, Danirixin and Reparixin respectively decreased the CSCs to 6.193 ± 0.875 % and 5.703 ± 0.419 % from 11.223 ± 0.738 %, however additional CXCL8 raised the CSCs to 14.040 ± 0.397 %. In SHP2 inhibited panel, Danirixin and Reparixin respectively decreased the CSCs to 1.807 ± 0.309 % and 1.970 ± 0.471 % from 3.757 ± 0.237 %, but additional CXCL8 increased the CSCs to 7.010 ± 0.920 %. **D** CXCL8-CXCR1/2 blockage limits the weight of SHP2 high PC9GR tumors than that of treated with DMSO, which decreased from 2.050 ± 0.184 g to 0.850 ± 0.023 g and 0.993 ± 0.070 g, and tumor weight SHP2 inhibited cells reduced from 1.576 ± 0.116 g to 0.510 ± 0.106 g and 0.474 ± 0.055 g, respectively. Each experiment was repeated 3 times

SHP2 might promote a positive CXCL8-CXCR1/2 feedback loop mediating tumor cell stemness and Osimeitinib resistance. To investigate this possibility, we treated parental and SHP2 modified PC9GR cells with CXCR2 inhibitor-Danirixin, CXCR1/2 inhibitor-Reparixin to mimic the blockage of the feedback loop, and human recombinant CXCL8 to enhance the feedback loop, respectively. The colony formation of SHP2 modified PC9GR cells were dramatically decreased by Danirixin or Reparixin, while exogenous CXCL8 significantly enhanced the colony formation of PC9GR (Fig. 6A). Furthermore, the percentage of CSCs was significantly reduced in the setting of CXCL8-CXCR1/2 loop blockage by Danirixin and Reparixin, and increased by recombinant CXCL8 enhancing the feedback loop (Fig. 6B). In SHP2 overexpressing panel, Danirixin and Reparixin respectively decreased the percentage of CSCs to 6.193 ± 0.875 % and 5.703 ± 0.419 % from 11.223 ± 0.738 %, while additional CXCL8 raised the CSCs to 14.040 ± 0.397 %. In SHP2 knock-down panel, Danirixin and Reparixin respectively decreased the CSCs to 1.807 ± 0.309 % and 1.970 ± 0.471 % from 3.757 ± 0.237 %, while additional CXCL8 increased the CSCs to 7.010 ± 0.920 %. We further found that CXCL8-CXCR1/2 blockage dramatically restrained the tumor generation of both SHP2 high and low expressing PC9GR cells *in vivo* (Fig. 6C). The tumor weight at the terminal point of the experiment confirmed the tumor curve, showing that CXCL8-CXCR1/2 blockage limit the weight of SHP2 overexpressing PC9GR tumors than those treated with DMSO, from 2.050 ± 0.184 g to 0.850 ± 0.023 g (Danirixin) and 0.993 ± 0.070 g (Reparixin), and the weight of SHP2 knock-down PC9GR tumors reduced from 1.576 ± 0.116 g to 0.510 ± 0.106 g (Danirixin) and 0.474 ± 0.055 g (Reparixin) (Fig. 6D). These data suggest that SHP2 mediates tumor cell stemness and tumorigenesis of PC9GR cells through a CXCL8/CXCR1 feedback loop.

## Discussion

Lung cancer is one of the most serious malignant diseases that imperil human health with leading incidences and mortality among all cancers around the world [1], and LUAD is the most prevalent histological subtype [2, 47]. Before targeted therapies are developed, poor outcome of 5-year disease free survival (DFS) and PFS, high recurrence and distant metastasis largely limited the clinical treatment and prognosis of LUAD [48, 49].

Identifying EGFR mutation as the driver oncogene was a significant milestone in the history of LUAD [50]. Mutant EGFR abnormally activates spontaneous downstream signaling pathways that promotes the malignant proliferation and metastasis of LUAD [51], and is also considered to be key reason for the failure of traditional radiotherapy and chemotherapy [52, 53]. Impressively, the prognosis of LUAD patients treated with first-generation EGFR-TKI was revolutionarily improved compared with chemotherapy, with the DFS increasing from 5.7 (5.2–6.3) months to 11.9 (9.1–14.6) months, and the OS improving from 24.3 (17.7–30.1) months to 25.8 (21.3–30.2) months, and the response rate increasing from 32.5 to 65.9 %. More interestingly, the EGFR-TKIs combination strategy could further enhance the PFS, OS and response rate to 17.5 months, 32.6 months and 82.5 %, respectively [53]. However, all LUAD patients inevitably developed resistance to 1st EGFR-TKIs within 8–14 months [54], resulting in the progression of the primary disease, of which 41–62 % cases due to an additional T790M mutation in EGFR [55]. The affinity of EGFR T790M mutation to ATP is dramatically higher than that to 1st EGFR-TKIs, and thus reduces the efficacy of first-generation drugs [56].

The 3rd generation EGFR-TKIs were designed to specifically bind EGFR with a T790M mutation, and offered a sufficient affinity that competitively suppress the ATP binding with the kinase domain [17]. In clinic, Osimertinib achieved a significant improvement on the outcome of 1st EGFR-TKIs resistant LUAD patients, which represents a great success of 3rd generation EGFR-TKIs. In newly diagnosed EGFR T790M LUAD patients, Osimertinib prolonged the median PFS to 8.2 (6.8–9.7) months, compared with patients who got 4.2 (4.1–5.1) months of PFS receiving platinum plus pemetrexed [57]. Osimertinib could even alleviate the disease progression in LUAD patients who failed 1st generation EGFR-TKIs treatment [58]. However, the current fundamental challenge is that around 66.43 % of patients acquire drug resistance after Osimertinib treatment, and current reports indicate that mutations in EGFR like T790M and C797S, KRAS mutations and novel gene fusions are possibly involved [18].

Recent data suggested that combination therapies based on the 3rd generation EGFR-TKIs could significantly improve the efficacy in LUAD clinic through enhancing sensitivity and reversing resistance [19, 20]. A phase II randomized trial indicated that an appropriate combination regime is especially important in this regard. Janne and colleges indicated that advanced EGFR mutant LUAD patients could not benefit from erlotinib and pemetrexed-cisplatin combined treatment [59].

Here, we found that SHP2 plays an important role in EGFR T790M mutant LUAD cells that are resistant to Osimertinib. SHP2 was enriched in Osimertinib resistant LUAD cells, and SHP2 inhibition enhances the killing effect of Osimertinib. We also confirmed that residual EGFR T790M mutant LUAD cells after treatment with Osimertinib expressed a high level of SHP2 protein, and SHP2 low-expressing LUAD cells were more sensitive to Osimertinib compared with SHP2 high-expressing cells both *in vitro* and *in vivo*. A synergistic effect in the suppression of proliferation of LUAD cells was observed when combining Osimertinib with the inhibition of SHP2, either through specific small molecule inhibitor or knock-down at the molecular level. These results suggest that blockage of SHP2 and T790M EGFR could potentially serve as a promising dual targeting strategy in LUAD. A few studies have actually shown that SHP2 inhibitors can synergistically promote the killing effect of several targeted drugs through overcoming the drug resistance [60–62]. For example, SHP2 inhibitors could reverse the sorafenib resistance of hepatocellular carcinoma by inhibiting MEK/ERK and AKT signaling [62]. Non-small cell lung cancer (NSCLC) patients with KRAS mutations and ALK rearrangement could benefit from SHP2 inhibitor combo therapy. Combination administration of SHP2 and MEK inhibitor can markedly suppress the proliferation of KRAS mutant NSCLC [63], and even reverse the drug resistance to first-generation EGFR-TKIs [64]. In ALK rearrangement NSCLC, SHP2 inhibitors combined with ALK inhibitors showed aggressive anti-tumor effect than a single drug *in vivo* and *in vitro*, and reversed the resistance of ALK inhibitors [65].

Next, we illustrated the potential mechanism of SHP2 in mediating the resistance of LAUD cells to Osimertinib. Multiple independent work reported that SHP2 regulates the maintenance of CSCs in various tissues [30, 63, 66–68], and the enrichment of stemness is often related to drug resistance of lung cancer [69, 70]. Similarly, Jiang et al. reported that SHP2 inhibitor restrained the expression of stem cell marker in KRAS mutant NSCLC, and the function of CSCs derived sphere was significantly inhibited [63]. Here we report for the first time that proliferation of EGFR T790M mutant LUAD cells is significantly enhanced by SHP2, and knock-down of SHP2 limits proliferation and enhances sensitivity to Osimertinib, and the stemness regulation by SHP2 derives from a CXCL8-CXCR1/2 feedback loop.

The mechanism of SHP2 regulating CSCs is mostly focused on the intracellular signaling, including but not limited to GSK3β-β-catenin [66], JNK [71] and Wnt [72]. CSCs play a vital role in regulating TME which is important to maintain the malignant proliferation of cancer cells [73–76]. Interleukins from TME could prime a positive feedback loop in maintaining the stemness of tumor [38, 77, 78]. We demonstrated here that SHP2 could promote tumor stemness via an interleukin feedback loop. We found 32 differentially expressed genes in SHP2 modified LUAD cells that clustered in secreted molecules, and CXCL8 is identified as the key mediator. The supernatant of SHP2 overexpressing EGFR T790M mutant LUAD cells contained a high concentration of CXCL8 than that of SHP2 low-expressing cells. Moreover, the percentage and biological function of CSCs was strengthened with exogeneous CXCL8 addition, and weakened when minimizing the CXCL8-CXCR1/2 loop through depleting endogenous CXCL8. CXCL8 is an important cytokine secreted by tumor cells that participates in initiating TME [79, 80], maintaining CSCs function [81–83], and leading drug resistance [84, 85]. Li et al. reported that SHP2 was positively correlated with the production of CXCL8 in acute cigarette smoke treated LUAD cells, and CXCL8 secretion decreased when SHP2 was inhibited [86]. Interestingly, CXCL8 was reported to induce the resistance of 1st generation of EGFR-TKI by promoting the stemness of LUAD cell line [87]. A clinic trial suggested that CSCs could be significantly reduced by inhibiting CXCR1/2 - CSCs labeled with ALDH1^+^ and CD44^+^/CD24^−^ decreased more than 20 % were monitored in 4/17 and 9/17 patients after administration of CXCR1/2 inhibitor [88]. Our results hereby conclude that SHP2 promotes stemness in EGFR T790M mutant LUAD cells through CXCL8-CXCR1/2 loop, a key mechanism mediating Osimertinib resistance. Furthermore, we report that this SHP2 regulated CXCL8-CXCL1/2 positive loop was mediated through an activated MAPK signaling. Consistent with our results, Mamik et al. reported that SHP2 promotes CXCL8 synthesis and secretion by activating p38 and ERK in MAPK pathway in astrocytes [89].

## Conclusions

**Fig. 7:**
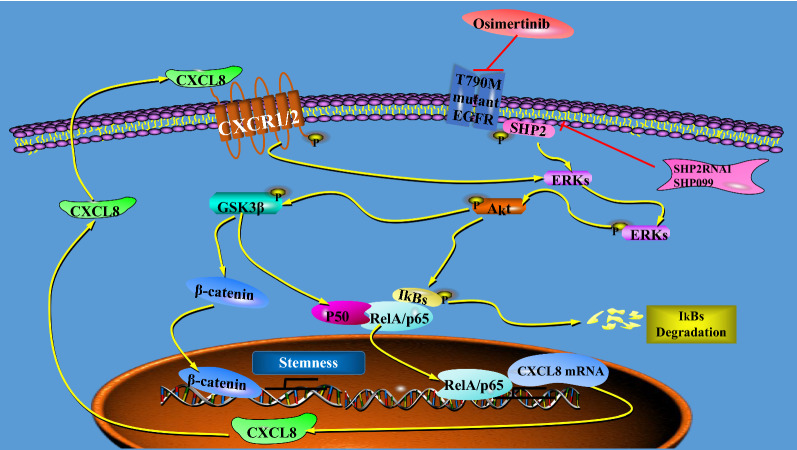
The role of SHP2 derived CXCL8/CXCR1 positive feedback loop in EGFR T790M mutant LUAD

We summarized the findings and mechanism from our experiments in Fig. 7. In brief, we found that inhibition of SHP2 in EGFR T790M mutant LUAD cells can improve the sensitivity of tumor cells to Osimertinib, and the mechanism is that inhibition of SHP2 suppresses the CSCs by blockade of CXCL8-CXCR1/2 positive feedback loop. This finding provides a molecular basis for a potential combined therapy of SHP2 inhibitor and Osimertinib to enhance the sensitivity of EGFR T790M mutant LUAD cells to Osimertinib and to minimize resistance. For sure, the safety and efficacy of a strategy that combines SHP2 inhibitor with Osimertinib in the treatment of EGFR T790M mutant or Osimertinib resistant LUAD patients have yet to be further verified by clinical data.

## Supplementary Information

**Additional file 1: Fig. S1. **High mRNA expression of genes in MEK-ERK and PI3K-AKT pathways was also associated with poor outcome of LUAD.

**Additional file 2: Fig. S2. **Quantification of Immunostaining results in Fig. 2C and F.

**Additional file 3: Fig. S3. **(A) In vitro tumor sphere were formed and CD133 + CSCs were analyzed by flow cytometry in SHP overexpressed and knock-down LUAD cells compared with their parental control cells at day 0 and day 7 of culture; (B) Transcriptome sequencing in SHP2 modified PC9GR cells and 1,203 variable genes across the datasets were identified for clustering which was highlighted the stem cell pathway; C and D, Pearson Correlation analysis and PCA analysis for the sequencing dataset in B, as quality control.

**Additional file 4: Tables S1. **In the tumorigenesis assay, the rate of tumor formation was recorded.

**Additional file 5: Tables S2. **CECL8 was highlighted in the 32 differentially expressed mRNA in inhibited, over-expressed and parental PC9GR cells in plot of secreted signaling molecule.

## Data Availability

transcriptome sequencing presented as Fig. 5A and Additional file 5: Tables S2 and made publicly available through GEO website.
