# Supplementary figures and images for: SHP2 inhibition enhances the anticancer effect of Osimertinib in EGFR T790M mutant lung adenocarcinoma by blocking CXCL8 loop mediated stemness
Source: Cancer Cell Int. 2021 Jul 3;21:337. doi: 10.1186/s12935-021-02056-x (PMC8254369; doi:10.1186/s12935-021-02056-x)

Supplementary Figure 1

Overall survival

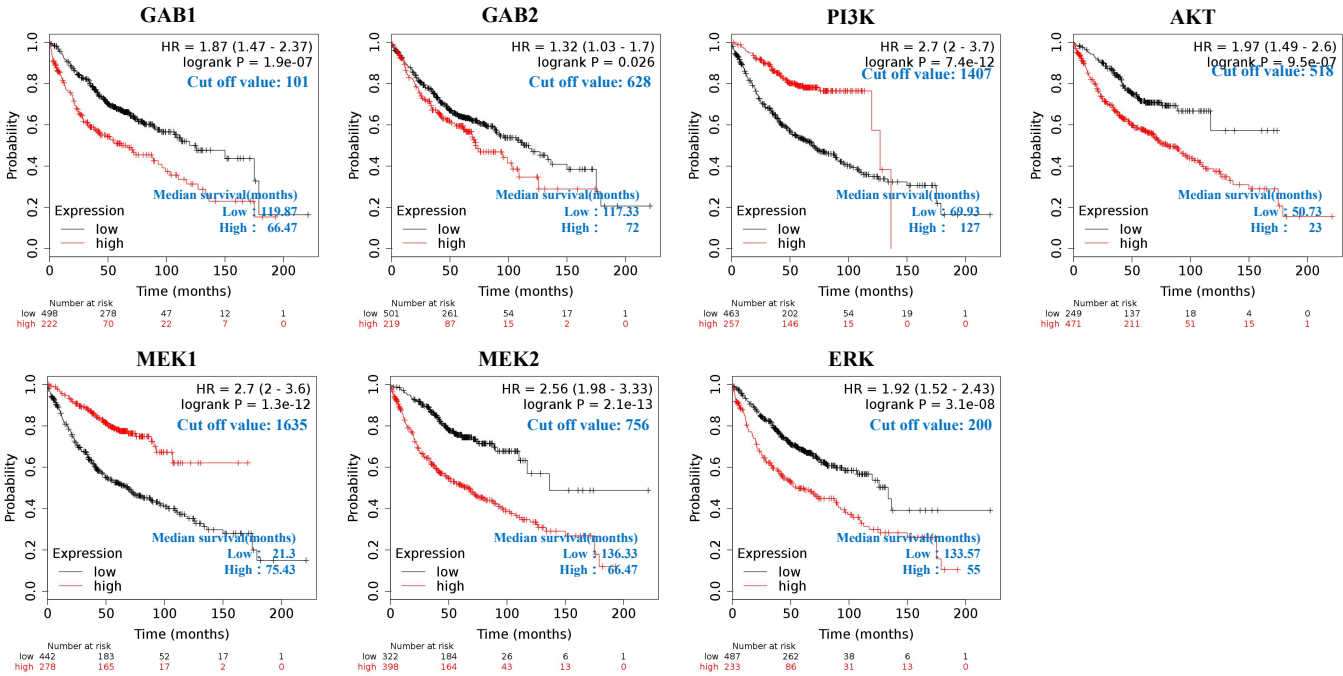

Progression free survival

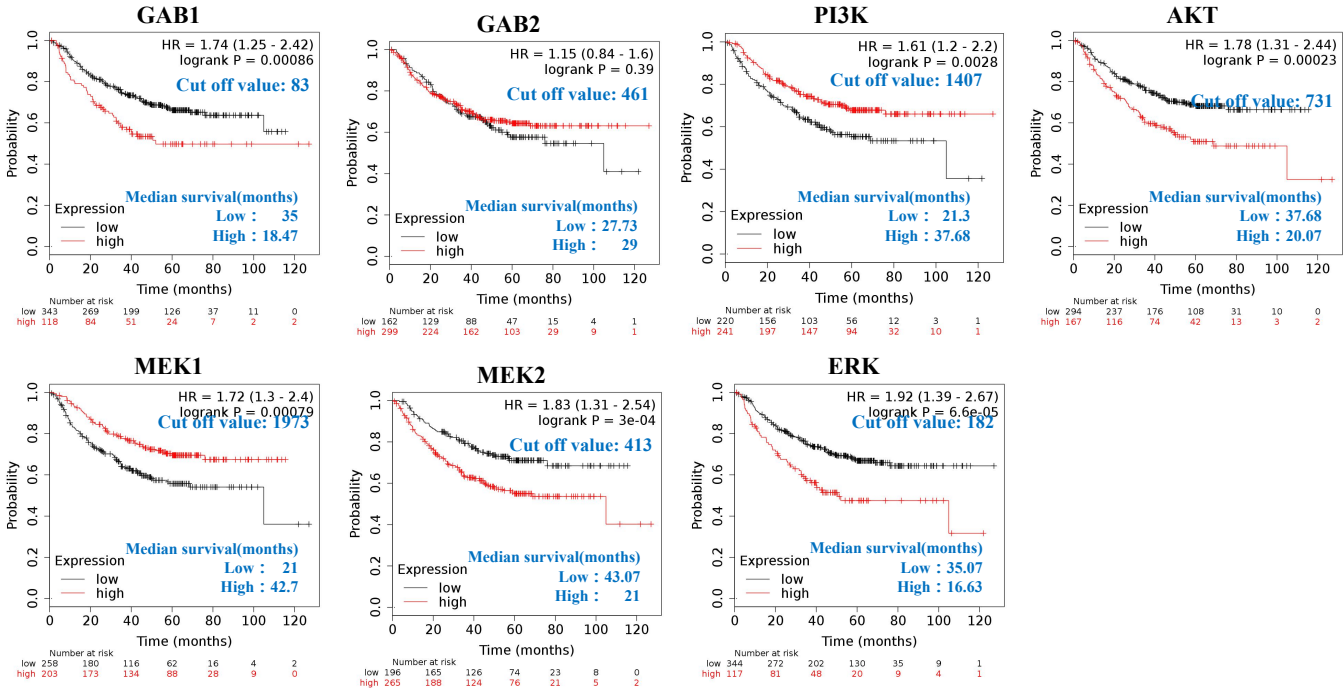

Supplement: Supplementary file 1 — Additional file 1: Fig. S1. High mRNA expression of genes in MEK-ERK and PI3K-AKT pathways was also associated with poor outcome of LUAD. [file 12935_2021_2056_MOESM1_ESM.pdf]

A

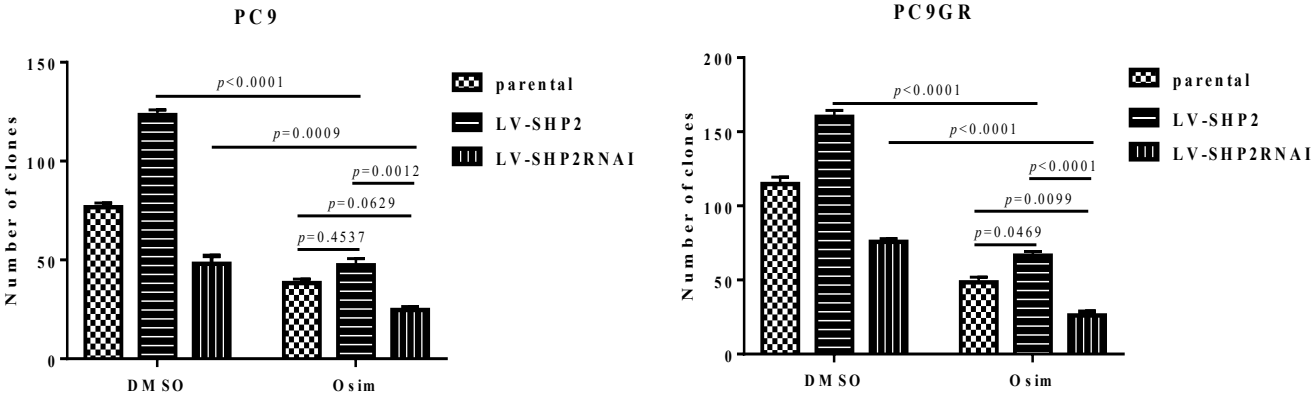

B

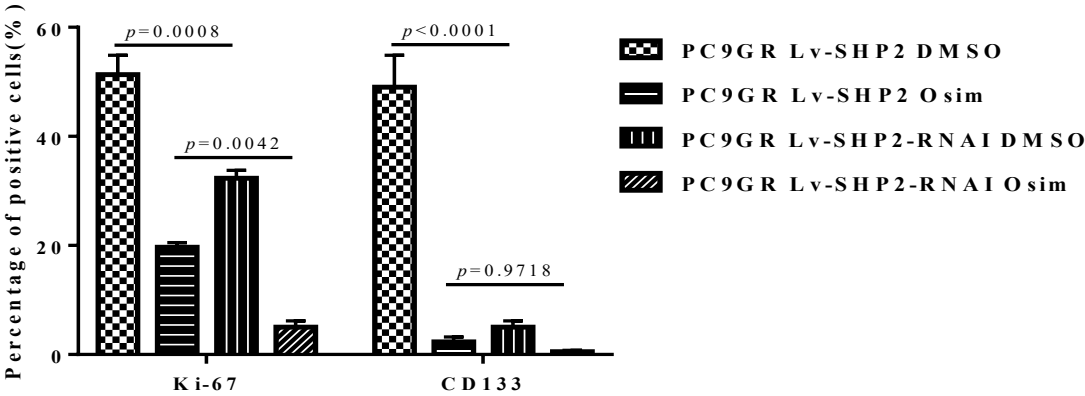

Supplement: Supplementary file 2 — Additional file 2: Fig. S2. Quantification of Immunostaining results in Fig. 2C and F. [file 12935_2021_2056_MOESM2_ESM.pdf]

Supplementary Figure 3

A

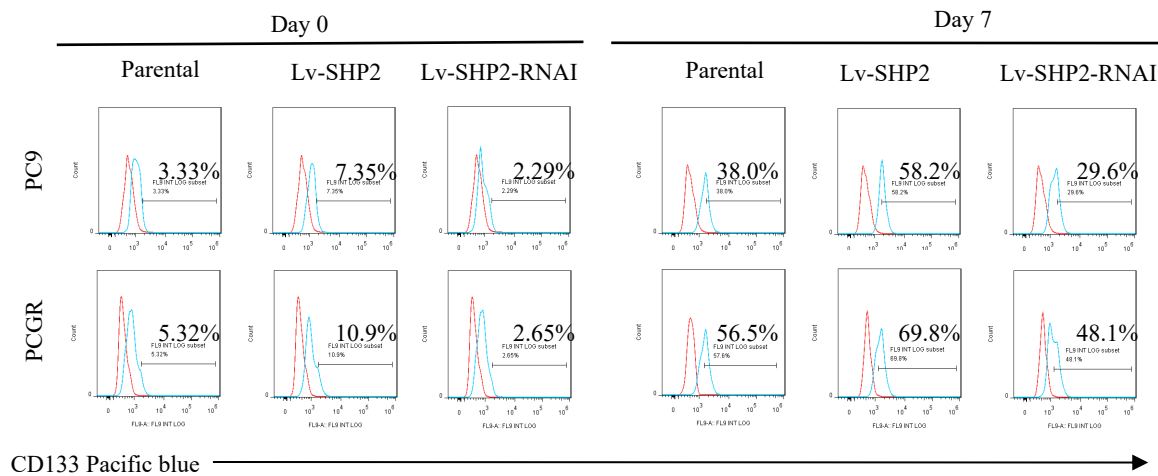

B

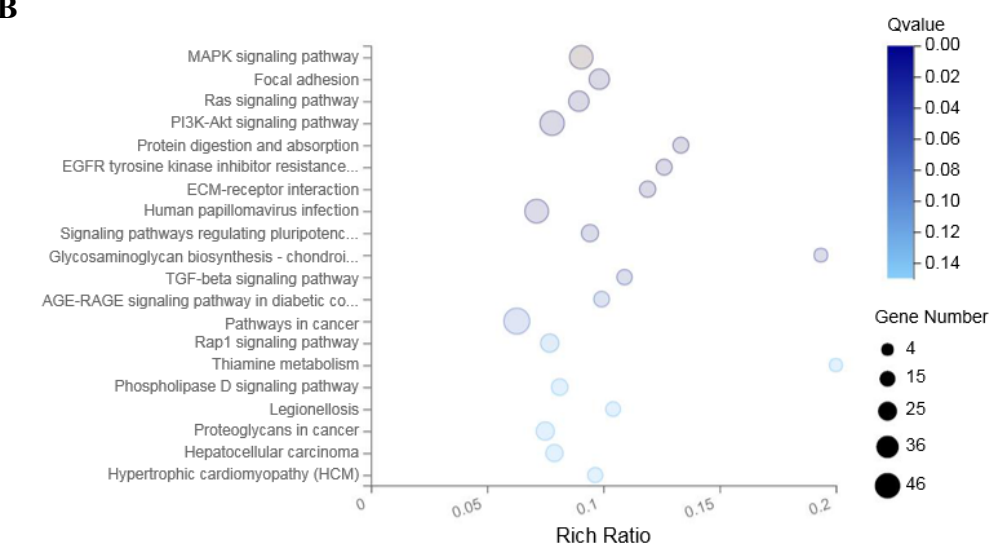

C

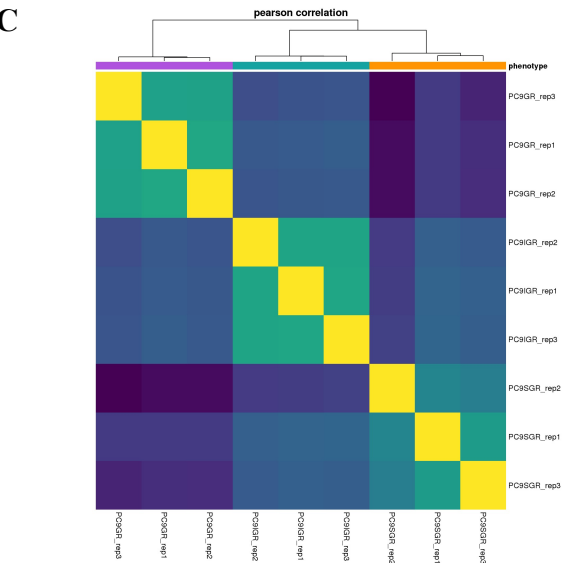

D

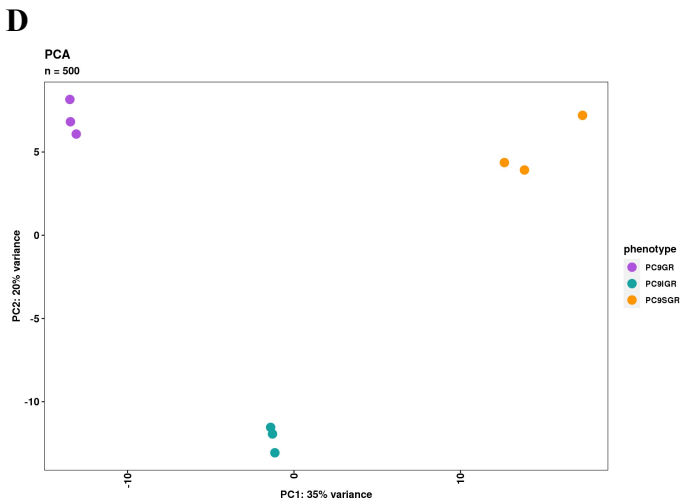

Supplement: Supplementary file 3 — Additional file 3: Fig. S3. (A) In vitro tumor sphere were formed and CD133 + CSCs were analyzed by flow cytometry in SHP overexpressed and knock-down LUAD cells compared with their parental control cells at day 0 and day 7 of culture; (B) Transcriptome sequencing in SHP2 modified PC9GR cells and 1,203 variable genes across the datasets were identified for clustering which was highlighted the stem cell pathway; C and D, Pearson Correlation analysis and PCA analysis for the sequencing dataset in B, as quality control. [file 12935_2021_2056_MOESM3_ESM.pdf]
