# Supplementary material for: SHP2 inhibition enhances the anticancer effect of Osimertinib in EGFR T790M mutant lung adenocarcinoma by blocking CXCL8 loop mediated stemness
Source: Cancer Cell Int. 2021 Jul 3;21:337. doi: 10.1186/s12935-021-02056-x (PMC8254369; doi:10.1186/s12935-021-02056-x)
Supplement: Supplementary file 4 — Additional file 4: Tables S1. In the tumorigenesis assay, the rate of tumor formation was recorded. [file 12935_2021_2056_MOESM4_ESM.pdf]

Cell density	PC9		PC9GR	
	Lv-SHP2	Lv-SHP2-RNAI	Lv-SHP2	Lv-SHP2-RNAI
50000	5/5	4/5	5/5	5/5
5000	5/5	3/5	5/5	4/5
500	2/5	0/5	3/5	1/5
