# Supplementary material for: SHP2 inhibition enhances the anticancer effect of Osimertinib in EGFR T790M mutant lung adenocarcinoma by blocking CXCL8 loop mediated stemness
Source: Cancer Cell Int. 2021 Jul 3;21:337. doi: 10.1186/s12935-021-02056-x (PMC8254369; doi:10.1186/s12935-021-02056-x)
Supplement: Supplementary file 5 — Additional file 5: Tables S2. CECL8 was highlighted in the 32 differentially expressed mRNA in inhibited, over-expressed and parental PC9GR cells in plot of secreted signaling molecule. [file 12935_2021_2056_MOESM5_ESM.pdf]

Gene symbol	gene ID	Parental			LV-SHP2-RNAI		
		1	2	3	1	2	3
IL-6	3569	0.09	0.31	0.29	0.09	0.07	0.12
VIP	7432	0.03	0.03	0	0	0.03	0
NCAM1	4684	0.01	0.03	0	0	0	0.01
GJB1	2705	0.06	0	0.03	0	0.03	0
DLGAP1	9229	0.19	0.25	0.08	0.32	0.18	0.19
NRP1	8829	3.93	3.49	3.9	3.57	3.79	3.01
CXCL8	3576	1.05	1.21	1.05	0.9	0.9	0.87
FGF11	2256	3.56	3.69	4.27	3.15	3.44	3.35
GJB2	2706	1.94	1.66	1.81	1.13	1.02	1.14
NAMP	10135	47.56	44.48	50.09	41.77	42.34	43.6
AREG	374	6.78	7.93	7.98	3.49	4.35	3.22
NGF	4803	4.69	4.15	5.32	5.75	6.87	6.68
ZYX	7791	54.47	54.29	54.93	58.19	62.23	57.08
GRB10	2887	2.05	1.92	1.75	2.3	2.59	2.39
ARL4D	379	2.09	2.08	2.32	2.94	3.03	2.39
STC2	8614	18.48	17.28	17.53	36.4	35.73	36.28
LTBP2	4053	4.31	4.37	4.05	6.54	6.64	6.71
CHRNB1	1140	11.31	12.2	12.48	14.87	15.25	15.38
IL1B	3553	1.3	1.2	0.93	0.68	0.29	0.65
EFNB2	1948	27.71	26.21	27.89	10.23	9.52	9.6
CTSE	1510	2.33	2.05	2.48	0.58	0.37	0.65
IL1A	3552	0.26	0.19	0.22	0.19	0.15	0.17
CXCL5	6374	0.04	0	0.04	0	0	0.02
MERTK	10461	2.63	0.99	2.04	0.65	0.72	1.46
GRAP2	9402	0.09	0.14	0.09	0.02	0.05	0.03
S100A6	6277	662.49	695.08	662.77	507.31	520.3	548.85
IL11	3589	2.16	2.34	2.2	0.68	0.86	0.64
EREG	2069	4.93	4.82	5.75	3.21	3.9	3.57
LIF	3976	14.07	14.14	14.17	10.14	10.55	9.82
PGC	5225	0.1	0.13	0.12	0.1	0.1	0.07
PTGIR	5739	3.19	8.46	7.01	1.5	6.64	6.81
CCL20	6364	0.11	0.22	0.26	0	0.23	0.06

	LV-SHP2	
1	2	3
0.43	0.9	0.61
0.03	0.12	0.1
0.01	0.06	0.01
0.06	0.21	0.03
0.33	1.21	0.38
4.71	4.72	4.57
1.77	2.08	1.97
9.78	9.95	10.84
4.36	4.37	4.56
59.66	59.68	56.65
16.48	15.57	16.14
16.22	14.95	16.25
85.42	85.69	84.93
11.31	11.78	11.53
3.94	4.68	3.55
51.73	50.81	50.37
10.03	10.43	9.98
19.72	20.98	19.4
0.65	0.91	0.85
20.75	21.25	20.3
0.9	1.13	0.85
0.26	0.22	0.29
0.04	0	0.09
1.69	1.16	2.51
0.08	0.14	0.12
721.42	758.8	656.27
2.53	2.48	2.53
6.39	6.16	6.02
15.89	18.05	17.73
0.07	0.18	0.24
7.91	6.46	11.12
0.23	0.29	0.53
